# Associations of physical activity with academic achievement and academic burden in Chinese children and adolescents: do gender and school grade matter?

**DOI:** 10.1186/s12889-022-13886-3

**Published:** 2022-08-05

**Authors:** Danqing Zhang, Jintao Hong, Sitong Chen, Yang Liu

**Affiliations:** 1 School of Physical Education, Shanghai University of Sport, Shanghai, 200438 China; 2 Shanghai Research Institute of Sports Science (Shanghai Anti-Doping Agency), Shanghai, 200030 China; 3 Institute for Health and Sport, Victoria University, Melbourne, VIC 8001 Australia; 4 Shanghai Research Centre for Physical Fitness and Health of Children and Adolescents, Shanghai, 200438 China

**Keywords:** Moderate-to-vigorous physical activity, Academic performance, Academic burden, Chinese school-aged students

## Abstract

**Background:**

Physical activity (PA) was significantly associated with cognition and mental health in children and adolescent. However, there were few studies examining the associations of PA with academic achievement (AA) and academic burden (AB) by gender and school grade. Hence, this study aimed to 1) investigate the associations of moderate-to-vigorous PA (MVPA) with AA and AB in Chinese children and adolescents, and 2) assess whether these associations vary by gender and school grade.

**Methods:**

Using a multi-stage stratified cluster sampling design (at four different regions in Southern east China), 2653 children and adolescents (8–19 years old, 51.2% girls) were included. A self-reported questionnaire was used to collect data on study participants’ gender, school grade, family social economic status (SES), parental education level, MVPA, AA and AB. Binary logistic regression was applied to examine the associations of MVPA with AA (groups: above-average AA, average and below-average AA) and AB (groups: reporting AB, reporting no AB) with odds ratios (ORs) and 95% confidence intervals (CIs). After testing gender*grade interaction, those associations were explored by gender and school grade separately.

**Results:**

In the overall sample, compared with children and adolescents who did not meet the PA guidelines (at least 60 min MVPA daily), children and adolescents who met the PA guidelines were more likely to have above-average (OR = 1.61, 95% CI: 1.21–2.11) AA, and report no AB (OR = 1.61, 95% CI: 1.13–2.30). In both genders, meeting the PA guidelines was positively associated with above-average AA (OR = 1.43, 95% CI: 1.01–2.03 for boys; OR = 2.22, 95% CI: 1.43–3.44 for girls). However, the significant relationship between meeting the PA guidelines and AB was observed only in girls (OR = 1.99, 95% CI: 1.17–3.39). Meeting the PA guidelines was positively associated with above-average AA (OR = 1.68, 95% CI: 1.18–2.40), and reporting no AB (OR = 1.77, 95% CI: 1.08–2.91) only in middle school students.

**Conclusions:**

This study suggested that sufficient PA may be a contributary factor of improved AA and lower level of AB in Chinese children and adolescents. However, associations of PA with AA and AB may be different across gender or school grade. Promoting PA among girls or middle school students may be a good approach to improve AA and reduce AB.

## Introduction

Academic achievement (AA) was referred to the ways that students accomplish specific goals, deal with their studies as well as the outcome of different academic assessment or examinations in school [1–3]. With school grade increasing, children and adolescents were expected to attain higher levels of AA, which may contribute to better career developments in the future. Hence, the importance of AA had been stressed for a long time [4]. China’s education institutions, like many countries, focused on strengthening education systems and enhancing students’ AA [5]. Thus, AA had been prioritized in Chinese social norm [6]. Enhanced AA was crucial for Chinese students since it was closely related to their entrance qualifications to university and a successful beginning of their future careers [7, 8].

School-aged students are usually suffering from heavy academic burdens (AB) for the reason that they need to do more academic assignments or relevant activities [9, 10]. AB refered to that students experience burden from academic outcomes including scores, success and expectations of external sources [11]. Studies suggested that academic learning is a main task for students [10], and AB was a complicated but extremely realistic issue that affects many aspects of school-aged students through the course of schooling education [12, 13]. Prior research suggests that high AB could lead to adverse effects on learning efficiency [14], health-related physiological and psychological outcomes in students [15]. Studies indicated that many children and adolescents in East Asian countries [16] had heavy academic loads [17]. It is not surprised that Chinese school-aged students are exposed to heavy AB in schools [9, 18], especially in higher grades and transitional schooling years [19].

Physical activity (PA) had been recognized having positive effects on children's and adolescents' health [20]. In particular, regular moderate-to-vigorous PA (MVPA) can lead to more additional health benefits, including lower risks of obesity, cardiovascular diseases and depression symptoms [21]. Moreover, one study showed that PA tracks from childhood to adulthood [22]. Physically active children and adolescents would be more likely to be active in later life, which in turn results in healthy lifestyle [23]. However, an increasing number of studies used nationally representative data showed that children and adolescents were in low levels of MVPA [24, 25]. In China, the situation was even worse. According to the Physical Activity and Fitness in China—The Youth Study (PAFCTYS), 86.9% of Chinese children and adolescents failed to meet the World Health Organization (WHO) recommendations for engaging at least 60 min MVPA per day (PA guidelines) [26, 27].

Previous studies showed that there were significantly positive relationships of PA with cognition, metacognition [28, 29] and mental health [30] in children and adolescents. Cognition skills were essential for school readiness, intelligence and AA [31–33] in children and adolescents. Systematic reviews and meta-analyses identified a positive relationship between PA and AA [1, 28], and most studies explored the association between MVPA and AA. One study found that self-report MVPA was positively associated with AA [34], while insufficient MVPA may be associated with worse memory [1] and poorer mental health [35]. However, according to prior research findings [36, 37], there was a weak relationship between PA and AA in students. Yet some studies reported inconsistent results [38–42], that was no associations [43, 44] or negative associations [45] between PA and AA. These discordant findings may be due to many reasons [1], for example, different effects by age [46]. Only a few studies had examined the age effects in the association between PA and AA, and the findings were inconsistent in older adolescents [41, 46]. In addition, based on Choi et al’s study [47], physically active adolescents were more likely to report less AB than inactive ones. The explicated empirical findings suggest that low PA may be a risk factor for AB. Recently, a study found that associations of PA with AA and mental health indicators in adolescents were both significant [48]. Further, previous studies demonstrated that age/gender differences [49] may be plausible reasons. Different developmental stages [50] or gender difference need to be emphasized in the future studies, as previous studies failed to consider gender or school grade differences [38].

It had been documented in exercise-brain model [51] and neurobiological mechanism [52, 53] hypothesis supported that participation in PA enhances cognition and mental health via changes in the structural and functional composition of the brain. Based on that, a hypothetical model for this study was constructed for PA, which would be associated with outcomes of significant changes in AA and AB among Chinses children and adolescents. To our knowledge, no study in China had examined associations of PA with AA and AB by gender and school grade. The purpose of this study were to 1) explore the associations of PA with AA and AB in Chinese children and adolescents, and 2) investigate gender and school grade differences in these associations.

## Methods

### Study design and participants

This study was a cross-sectional survey using a multi-stage stratified cluster sampling design. Initially, we recruited school-aged students in one municipality (Shanghai) and three provinces (Anhui, Zhejiang and Jiangsu). Seven primary schools, ten middle and high schools from Shanghai, six middle and high schools from Anhui, four middle and high schools from Zhejiang, and six middle and high schools from Jiangsu were conveniently recruited. In each school, study participants of at least one class in one grade was selected randomly. Then, all students in the class were included for survey. In total, 3111 students (3-11th grades, aged 8–19 years, 52.8% girls) were selected and consented to take part in this study survey. After excluding invalid cases with missing information or answers, 2653 (51.2% girls) participants completed the questionnaire survey (response rate: 85.3%). The permission to conduct this study was obtained from the teachers and principals of those participating schools. All children and adolescents participating in the study, and their parents or guardians, were informed and participants were voluntary to take part in this survey. This study survey was approved by Institution of Review Board at Shanghai University of Sport. Data were collected and analyzed anonymously.

### Procedures

Prior to the study survey, all participants were informed with the aim of the study. Research staff provided a detailed description of the survey and instruct children and adolescents how to complete a 3-page paper-based questionnaire that included questions about their MVPA, AA, AB and other sociodemographic information. Trained research staff administered the survey and help to answer any question when study participants have any problems to understand the survey.

### Measurements of variables

#### Study outcome (Self-report AA)

Self-reported AA had been confirmed as an acceptable measurement in previous research [34, 54]. In this study, Children and adolescents’ AA was measured via a reliability question [55] of HBSC questionnaire. A 2-week interval test–retest reliability of AA was assessed by the ICC on 95 Chinese school-aged students, with ICC of 0.58. Self-reported AA question was *“In your opinion, what does/do your class teacher(s) think about your school performance compared to your classmates?”* and the participant need to select one response ranging from *“very good, good, average, below average”*, and correspond on a 4-point scale, they self-reported their own average AA level based on the following scores: 4 = “very good”, 3 = “good”, 2 = “average”, 1 = “below average” in the class, with high scores indicating outstanding AA. On the basis of their responses [34], the participants were divided into two groups: 1) those who reported “good” and “very good” AA (above-average AA, coded = 1) and 2) those who reported average and below average AA (coded = 0).

#### Study outcome (Self-report AB)

Self-reported AB was evaluated by *“How much burden do you feel by studying recently?”* and the participant was told to pick one response ranging from *“much, some, a little, none”*, and correspond on a 4-point scale, they self-reported their own average AB level based on the following scores: 4 = “much”, 3 = “some”, 2 = “a little”, 1 = “none” about their study, with high level indicating AB was particularly heavy. The measure applied in HBSC survey had been confirmed as reliable in previous researches [12, 56]. A 2-week interval test–retest reliability of AA was assessed by the ICC on 95 Chinese school-aged students, with ICC of 0.62. On the basis of their responses, the participants were divided into two groups: 1) those who reported none AB (no AB, coded = 1) and 2) those who reported a little, some, much AB [9] (having AB, coded = 0).

#### Study exposure (Self-report MVPA)

Children and adolescents’ MVPA was measured by questions from the Health Behavior in School-Aged Children (HBSC) survey questionnaire [57] which were reliable and validated in school-aged students [58, 59]. Children and adolescents reported the average daily minutes of joining in MVPA in the past week. Self-reported MVPA were evaluated by two questions (days of MVPA on weekdays and weekend days, respectively) [26, 60, 61]. This modified version were assessed by 2-week interval test–retest reliability of the intraclass correlation coefficient (ICC) on 107 school-aged students, with ICC of 0.53 and 0.52 respectively. The first question was *“During the previous five weekdays, on how many days were you physically active for a total of at least 60 min per day?”* and children and adolescents were asked to select a response ranging from zero days to five days (*responses: 0* = *0 day, 1* = *1 day, 2* = *2 days, 3* = *3 days, 4* = *4 days, 5* = *5 days*). The second question was *“During the previous two weekend days, on how many days were you physically active for a total of at least 60 min per day?”* and children and adolescents were required to choose a response ranging from zero days to two days (*responses: 0* = *0 day, 1* = *1 day, 2* = *2 days*). To help participants better understand MVPA, it was explained as *“any kind of PA that increased your heart rate and made you breathe hard some of the time (including physical education time, exercising, sports training, and various regular daily activities such as brisk walking, hiking, and excursion)”* [60]. We aggregated the responses from these two questions to compute the total number of days those children and adolescents were physically active for at least 60 min [26, 60]. We also corresponded and calculated the overall percentage of students who met the PA guidelines of being physically active for at least 60 min daily [27, 62]. Additionally, the participants were divided into two groups: 1) those who reported meet the PA guidelines (coded = 1) and 2) those who did not meet [63](coded = 0).

#### Self-report socio-demographic covariates (Other measures)

Demographic information included children and adolescents’ gender, school grade, social economic status (SES) [64] and parental education level [64] for each children and adolescents was obtained through his or her self-report on the survey. Participants were required to report their information on gender (*responses: 1* = *male, 2* = *female*), grade (*responses: 1, 2, 3, … 12*), SES (*responses: 1* = *very good, 2* = *good, 3* = *average, 4* = *poor, 5* = *very poor*), and parental education level was separately assessed by asking mother and father’s education level (*responses: 1* = *under primary school, 2* = *primary school, 3* = *middle school, 4* = *high school, 5* = *college, 6* = *university bachelor, 7* = *university master or higher*). Based on actual situation of basic education in China, grades were divided of three school grade groups: Primary (1-6th grade, shanghai:1-5th grade), middle (7-9th grade, shanghai:6-9th grade) and high (10-12th grade) schools.

### Statistical analysis

All the statistical analysis were performed in SPSS Version 25.0 (IBM Corp., Armonk, NY, USA). Descriptive analysis was used to reported characteristics (mean or percentage) of variables of this study. Chi-square test was used to examine the difference among MVPA, AA or AB by gender or school grades. Binary logistic regression was applied to estimate the associations among MVPA with AA and AB after adjusting for all the sociodemographic variables (gender, school grades, SES, and parental education level) with odds ratios (ORs) and 95% confidence intervals (CIs). As a gender*grade interaction role was found in the associations of MVPA with AA and AB, we did stratified analysis by gender and school grades separately. Statistical significance was set at *p* < 0.05.

## Results

### Participant’s characteristics

Characteristics of the overall study participants were presented in Table 1. In total, 2653 children and adolescents (1359 girls, 51.2%) were included, with a mean age of 13.0 years (Standard Deviation, SD = 2.4). Over 68.0% of children and adolescents reported above-average AA and only 9.9% of children and adolescents reported no AB. Moreover, only 10.7% of study participants met the PA guidelines.

**Table 1:** Characterization of children and adolescents

Variables		Total (*n* = 2653)	Boys (*n* = 1294, 48.8%)	Girls (*n* = 1359, 51.2%)
*Age*		13.0 ± 2.4	12.8 ± 2.3	13.2 ± 2.4
*School*				
Primary		472 (17.8%)	240 (18.5%)	232 (17.1%)
Middle		1711 (64.5%)	885 (68.4%)	826 (60.8%)
High		470 (17.7%)	169 (13.1%)	301 (22.1%)
*Parents’ education level*				
Under primary school		42 (1.6%) ^F^, 113 (4.3%) ^M^	23(1.8%) ^F^, 61 (4.7%) ^M^	19 (1.4%) ^F^, 52 (3.8%) ^M^
Primary school		234 (8.8%) ^F^, 361 (13.6%) ^M^	109(8.4%) ^F^, 179 (13.8%) ^M^	125 (9.2%) ^F^, 182 (13.4%) ^M^
Middle school		802 (30.2%) ^F^, 773 (29.1%) ^M^	384(29.7%) ^F^, 357 (27.6%) ^M^	418 (30.8%) ^F^, 416 (30.6%) ^M^
High school		612 (23.1%) ^F^, 481 (18.1%)	310(24.0%) ^F^, 239 (18.5%) ^M^	302 (22.2%) ^F^, 242 (17.8%) ^M^
College		299 (11.3%) ^F^, 331 (12.5%) ^M^	145 (11.2%) ^F^, 165 (12.8%) ^M^	154 (11.3%) ^F^, 166 (12.2%) ^M^
University bachelor		471 (17.8%) ^F^, 448 (16.95) ^M^	215 (16.6%) ^F^, 211 (16.3%) ^M^	256 (18.8%) ^F^, 237 (17.4%) ^M^
University master or higher		193 (7.3%) ^F^, 146 (5.5%) ^M^	108 (8.3%) ^F^, 82 (6.3%) ^M^	85 (6.3%) ^F^, 64 (4.7%) ^M^
*SES*				
Very good		80 (3.0%)	54 (4.2%)	26 (1.9%)
Good		773 (29.1%)	393 (30.4%)	380 (28.0%)
Fair		1525 (57.5%)	716 (55.3%)	809 (59.5%)
Poor		226 (8.5%)	107 (8.3%)	119 (8.8%)
Very poor		49 (1.8%)	24 (1.9%)	25 (1.8%)
*MVPA*				
0 days		141 (5.3%)	56 (4.3%)	85 (6.3%)
1 days		202 (7.6%)	86 (6.6%)	116 (8.5%)
2 days		299 (11.3%)	127 (9.8%)	172 (12.7%)
3 days		465 (17.5%)	237 (18.3%)	228 (16.8%)
4 days		558 (21.0%)	266 (20.6%)	292 (21.5%)
5 days		353 (13.3%)	165 (12.8%)	188 (13.8%)
6 days		351 (13.2%)	179 (13.8%)	172 (12.7%)
7 days		284 (10.7%)	178 (13.8%)	106 (7.8%)
*Academic achievement*				
Very good		118 (4.4%)	71 (5.5%)	47 (3.5%)
Good		709 (26.7%)	359 (27.7%)	350 (25.8%)
Average		1566 (59.0%)	720 (55.6%)	846 (62.3%)
Below average		260 (9.8%)	144 (11.1%)	116 (8.5%)
*Academic burden*				
Much		444 (16.7%)	237 (18.3%)	207 (15.2%)
Some		1028 (38.7%)	497 (38.4%)	531 (39.1%)
A little		919 (34.6%)	427 (33.0%)	492 (36.2%)
None		262(9.9%)	133 (10.3%)	129 (9.5%)

Notes: *MVPA* Moderate-to-Vigorous Physical Activity: number of days that children and adolescents are physically active for at least 60 min, *SES* social economic status, ^F^: Father’s education level, ^M^: Mother’s education level

Table 2 showed differences of prevalence of meeting the PA guidelines, AA and AB by gender or school grade. In details, more boys reported above-average AA (33.2% vs. 29.2%, *p* < 0.05) and were more physically active than girls (13.8% vs. 7.8%, *p* < 0.001). Differences were found in prevalence of meeting the PA guidelines, AA, AB across different school grade (*p* < 0.001). With school grade increasing, the percentage of meeting the PA guidelines, above-average AA and reporting no AB decreased.

**Table 2:** The difference in variables by gender or school grade

	Total (*n* = 2653)	Gender		School grade		
		Boys (*n* = 1294)	Girls (*n* = 1359)	Primary (*n* = 472, 9.1 ± 0.9ys)	Middle (*n* = 1711, 13.2 ± 1.3ys)	High (*n* = 470, 16.0 ± 0.7ys)
*MVPA*						
Meet the PA guidelines	284 (10.7%)	178 (13.8%)	106 (7.8%)	91 (19.3%)	165 (9.6%)	28 (6.0%)
Not meet the PA guidelines	2369 (89.3%)	1116 (86.2%)	1253 (92.2%)	381 (80.7%)	1546 (90.4%)	442 (94.0%)
		*p* < 0.001		*p* < 0.001		
*AA*						
Above-average	1826 (68.9%)	430 (33.2%)	397 (29.2%)	237 (50.2%)	490 (28.6%)	100 (21.3%)
≤ Average	827 (31.1%)	864 (66.8%)	962 (70.8%)	235 (49.8%)	1221 (71.4%)	370 (78.7%)
		*p* < 0.05		*p* < 0.001		
*AB*						
No AB	262 (9.9%)	133 (10.3%)	129 (9.5%)	111 (23.5%)	137 (8.0%)	14 (3.0%)
Having AB	2391 (90.1%)	1161 (89.7%)	1230 (90.5%)	361 (76.5%)	1574 (92.0%)	456 (97.0%)
		*p* = 0.50		*p* < 0.001		

Notes: *MVPA* Moderate-to-Vigorous Physical Activity. PA guidelines (Physical Activity guidelines): World Health Organization recommendation for at least 60 min MVPA daily. Above-average AA: above-average academic achievement (included good and very good), AB: academic burden. ys: years old

### Associations of self-report MVPA with AA and AB

Binary logistic regression analysis revealed associations of MVPA with AA and AB in the overall study participants. Compared with those who did not meet the PA guidelines, children and adolescents who met the PA guidelines had a greater likelihood of reporting above-average AA (OR = 1.61, 95% CI: 1.23–2.11). Similar results were also observed the significantly relationships between MVPA and AB in children and adolescents (OR = 1.61, 95% CI: 1.13–2.30).

Table 3 presented the results for associations of MVPA with AA and AB among boys and girls. Compared with those who did not meet the PA guidelines, boys who met the PA guidelines have greater likelihood of attaining above-average AA (OR = 1.43, 95% CI: 1.01–2.03). Similar results were also observed among girls (OR = 2.22, 95% CI: 1.43–3.44). Compared with those who did not meet the PA guidelines, girls who met the PA guidelines have greater likelihood of reporting no AB (OR = 1.99, 95% CI: 1.17–3.39).

**Table 3:** Associations of MVPA with AA and AB by gender

	Total (*n* = 2653)		Boys (*n* = 1294)		Girls (*n* = 1359)	
	OR	95% CI	OR	95% CI	OR	95% CI
AA						
Not meet the PA guidelines	1		1		1	
Meet the PA guidelines	**1.61**	**1.23–2.11**	**1.43**	**1.01–2.03**	**2.22**	**1.43–3.44**
AB						
Not meet the PA guidelines	1		1		1	
Meet the PA guidelines	**1.61**	**1.13–2.30**	1.44	0.88–2.33	**1.99**	**1.17–3.39**

Notes: *OR* Odd Ratio, *CI* Confidence Interval, *PA* Physical Activity, *AA* Academic achievement, *AB* Academic burden. *p* < 0.05 tested by Binary logistic regression analysis (adjusted for grades, gender, parents’ education level and SES). 1 = Reference

As was shown in Table 4, results for associations of MVPA with AA and AB by school grade. Compared with those who did not meet the PA guidelines, children and adolescents who met the PA guidelines may be significantly associated with above-average AA (OR = 1.68, 95% CI: 1.18–2.40) and reporting no AB (OR = 1.77, 95% CI: 1.08–2.91) in middle school.

**Table 4:** Associations of MVPA with AA and AB by school grade

	Primary school (*n* = 472, 9.1 ± 0.9ys)		Middle school (*n* = 1711, 13.2 ± 1.3ys)		High school (*n* = 470, 16.0 ± 0.7ys)	
	OR	95% CI	OR	95% CI	OR	95% CI
AA						
Not meet the PA guidelines	1		1		1	
Meet the PA guidelines	1.53	0.92–2.52	**1.68**	**1.18–2.40**	1.68	0.70–4.04
AB						
Not meet the PA guidelines	1		1		1	
Meet the PA guidelines	1.70	0.99–2.93	**1.77**	**1.08–2.91**	3.28	0.45–24.11

Notes: *OR* Odd Ratio, *CI* Confidence Interval, *PA* Physical Activity, *AA* Academic achievement, *AB* Academic burden. *p* < 0.05 tested by Binary logistic regression analysis (adjusted for grades, gender, parents’ education level and SES). ys: years old. 1 = Reference

## Discussion

### Study findings

This study focused on investigating associations of MVPA with AA and AB in Chinese children and adolescents. In addition, this study also explored gender and grade differences in the above associations. Results showed that approximately one in tenth of children and adolescents met the PA guidelines, with boys being more physically active than girls. Moreover, with grade increasing, children and adolescents were less likely to meet the PA guidelines. Compared with children and adolescents who did not meet the PA guidelines, those who met the PA guidelines were more likely to have above-average AA and report no AB. In general, MVPA may be positively associated with AA in both genders. However, the association between MVPA and AB was differentiated in boys and girls. Meeting the PA guidelines may be positively associated with AA and AB in middle school students rather than students in other school grade.

### PA and AA

This study pointed that Chinese children and adolescents who met the PA guidelines were more likely to report above-average AA, compared with those who did not meet the PA guidelines. These results were consistent with other previous findings [39, 48, 65, 66]. Some possibly direct explanations can be used to support the positive association between MVPA and AA in this study. Participating in PA may enhance both health and function of brain [67–69] among children and adolescents. For health of brain, the production of new nerve cells in hippocampal stem cells [51] may increase by engaging in PA. For function of brain, arousal induced and boredom can reduce by PA, thus the attention span increased [70]. Moreover, doing PA may benefit cognitive functions, such as working memory and information processing in learning, thereby the integration and distribution of academic information may expedite of youth [71–73]. Additionally, several indirect reasons were explainable. Participation in PA was possibly linked to learning defined as improved self-concept and increased academic self-confidence [74]in children and adolescents. Another study observed that the level of self-esteem may be enhance and future occupational outcomes may be affected by participating in PA [75], which was consistent with findings that PA benefits diverse academic-related outcomes in children and adolescents [76]. However, the association would be bidirectional due to cross-sectional study. Insights could be gained from conducting high-quality PA intervention on AA among Chinese children and adolescents.

### PA and AB

This study showed that those who met the PA guidelines may be nearly two times greater to have no AB than those who did not meet the PA guidelines. This finding was supported by a prior study [77], which MVPA had been shown to be linked to physical and psychosocial health outcomes of children and adolescents. Promoting PA could affect circulatory system in the whole body [78]. For example, blood flow and ions can be improved by participation in PA, and thus hormones may be mobilized, which in turn enhances blood circulation. Thus, PA can lead to positive changes in psychological emotion [79] in children and adolescents. In addition, PA benefited mental or psychosocial health. Landers & Petruzzello’ study showed that PA may be anxiolytic and antidepressant [80]. On the contrary, lower levels of PA may be associated with depressive symptoms [81]. Meanwhile, Lima et al’ findings showed that a strong correlation between depression and burden [82]. This relationship may indirectly strengthed the association of MVPA and AB in children and adolescents. Regardless of the direct or indirect associations between PA and mental health, physically inactive children and adolescents may suffer a ‘double health burden’, with both physical and mental health challenges [50]. However, since cross-sectional study cannot infer bidirectional associations, this study suggested PA intervention on AB can be a way of confirming the relationship in future research.

### PA with AA and AB by gender

This study may indicate that the associations of MVPA with AA and AB varied by gender. The evidence showed that girls may have lower AA levels than boys. It is possible that higher levels of MVPA had a more profound effect on AA in girls. Moreover, gender differences in the association between MVPA and AA may be owing to different school subjects. For instance, girls may have greater ability in learning Language, Literature, Biology, Geology, and Plastic, while boys outperform Mathematics, Physical Education, and Technology [83, 84]. This might lead to the varied associations. Additionally, this study also found that the negative association between MVPA and AB was not significant in boys but girls. Prior research showed that individuals regularly engaging in organized and team sports were associated with the lower risks of mental health problems [30]. Yet the potential benefits in organized and team activities with mental health may be different in boys and girls. One study reported that organized sports had a greater impact on mental health in girls than boys [85]. It is possible that team sports lead to mental health benefits for girls particularly [86]. In the future, it is important to provide more evidence supporting the gender difference in the association of MVPA with AA and AB.

### PA with AA and AB by school grade

This study found that only middle school students who met the PA guidelines were more likely to have above-average AA and reporting no AB compared with those who did not meet the PA guidelines. There were several possible reasons to explain this research finding. Firstly, this result may be due to education System in China. Primary (1-6^th^ grade, shanghai:1-5^th^), middle (7-9^th^ grade, shanghai:6-9^th^) and high (10-12^th^ grade) schooling are parts of basic education in China, with the middle school grade being a demarcation point between compulsory education (1-9^th^ grade) and non-compulsory education (10-12^th^ grade). During compulsory education period, students were enrolled to the nearest school according to their residence area, which had nothing to do with their AA. However, in order to continue their study in high school, students have to attend entrance examination when graduated from middle school. Only about half of students can go to high school based on their scores of entrance examination. The other students may enter vocational or occupational school. For those students who can continue studying in high school, the score of entrance examination was also vital for choosing schools. The higher scores they have, the better high school they can be enrolled. One study showed that Chinese parents were reputed to have high educational expectations of their children school achievement [87]. Therefore, AA was important in middle school students, and cause their AB to be highly concerned. Moreover, promoting MVPA of children and adolescents, especially middle school students, were committed by Chinese government and education agency. Recently, it suggested that physical education subject will receive sufficient attention as equal as subjects of Chinese, mathematics and languages. Thus, PA opportunity will be increased greatly in school education system in China. Secondly, middle school students were in pubertal spurt phase and this stage was a critical period for their overall growth. The physical changes occur alongside mental, cognition and movement development changes [88] in children and adolescents. Explanations for why adolescents participated in adequate MVPA may have greater importance in reducing psychological distress and enhancing cognition skills. It may also be related to how peer support and interaction play an increasingly important role [88, 89] in childhood, which may modified the associations of MVPA with AA and AB. Thirdly, this findings may be due to various reasons [1], including sample size in different school grades and individual differences of children and adolescents. The sample size of primary school and high school was small, which was one of the possible reasons for the relationship. Finally, this study supported previous study findings that no significant association was found between MVPA and AA in younger school-age children [44, 90, 91]. However, findings of this study was inconsistent with the latest summary findings that MVPA has both short- and long-term positive effects on brain health, cognitive functions and academic performance in 5–13 years old children [92]. The possible reason for incompatible findings that the summary findings were mainly based on western children and adolescent, which may not be replicated in China. In addition, the summary finding also showed that no significant association between MVPA and AB in high school students. Previous studies had shown that, during high school years, some adolescents were under heavy pressure in school and heavy homework loads were typical [5, 93]. It was therefore that many high school students do not have enough time for MVPA [9]. Those factors may explain the significant association of MVPA with AA and AB observed in middle school students.

### Strengths and limitations

Strengths of this study include that exploration on associations of MVPA, a specific intensity of PA with strongly benefits for physical and mental health, with AA and AB in Chinese children and adolescents by gender and school grade. Moreover, in this study, the sample size was large and students are recruited from different regions, which may increase the generalizability of research findings. However, some limitations were involved in this study. Firstly, the sample size of primary and high school grades was small, which may have an impact on the research findings. Secondly, this study used self-reported questionnaires to assess MVPA, SES, parents’ education, AA and AB in children and adolescents, which may impact the accuracy of measurement. Thirdly, children and adolescents’ BMI which may influence independent and dependent variables was not included, and it could lead to bias to results. Moreover, some confounding factors (such as, learning difficulties, the need for educational support, physical disabilities) for PA, AA, and AB were not measured and it may not be possible to exclude the influence of these factors. Finally, only associations of MVPA intensity with AA and AB are included and limitation of cross-sectional study designs. Hence, PA intervention on AA and AB in Chinese children and adolescents needs to be clarified in the future research.

## Conclusion

PA may be significantly associated with AA and AB in Chinese children and adolescents, especially in girls. MVPA was probably associated with AA and AB in middle school adolescents. Promoting girls or middle school students PA may significantly influence their AA and AB. Further studies may be encouraged to explore associations of different intensity of PA with AA and AB in Chinese children and adolescent by different gender and school grade. Moreover, more interventions, longitudinal or mediation studies may be needed to clarify relationships of PA with AA and AB.

## Data Availability

The datasets analysed in this study are available from the corresponding author on reasonable request.

## Abbreviations

CI: Confidence interval
OR: Odds ratio
PA: Physical activity
MVPA: Moderate-to-vigorous physical activity
AA: Academic achievement
AB: Academic burden
SES: Social economic status
WHO: World Health Organization
